# Genome-Wide Association Study of Body Size Traits in Luning Chickens Using Whole-Genome Sequencing

**DOI:** 10.3390/ani15070972

**Published:** 2025-03-27

**Authors:** Zhiyi Li, Yi Nong, Yuan Liu, Zi Wang, Jiayan Wang, Zhixiong Li

**Affiliations:** 1 Key Laboratory of Qinghai-Tibetan Plateau Animal Genetic Resource Reservation and Utilization of Ministry of Education, Southwest Minzu University, Chengdu 610041, China; daniellizhiyi009@gmail.com (Z.L.); nongyi1211@163.com (Y.N.); yuan_liu59@163.com (Y.L.); ziw44032@gmail.com (Z.W.); wangjiayantcwx@163.com (J.W.); 2 Key Laboratory of Animal Science of National Ethnic Affairs Commission of China, Southwest Minzu University, Chengdu 610041, China; 3 Institute of Qinghai-Tibetan Plateau, Southwest Minzu University, Chengdu 610041, China

**Keywords:** chicken, GWAS, body traits, genetic variation, SNPs

## Abstract

Growth traits are crucial for poultry breeding and production. Marker-assisted selection (MAS) and genomic selection (GS) of growth traits require a substantial number of accurate genetic markers. A genome-wide association study (GWAS) for body size traits was performed on 248 Luning chickens to identify significant single-nucleotide polymorphisms (SNPs) and insertions and deletions (INDELs) related to the growth and development of chickens. A total of 30 significant SNPs and 13 INDELs were obtained for body size traits. Two notable regions, spanning from 43.072 to 43.219 Mb on chromosome 1 and from 4.751 to 4.800 Mb on chromosome 11, were found to be significantly associated with growth traits in the GWAS of both SNPs and INDELs. Some genes, including *PPFIA2*, *KITLG*, *DUSP6*, *TOX3*, *MTNR1B*, *FAT3*, *PTPRR*, *VEZT*, *BBS9*, and *CYLD*, were identified as important candidate genes for the growth of chickens. The results provide valuable information for understanding the genetic basis of growth traits which is beneficial for the subsequent selective breeding in Luning chickens.

## Simple Summary

With the development of breeding technology, the broiler industry has significantly evolved. All the yellow-feather broilers used in production in China are developed from local chicken breeds. As an underutilized genetic resource, the Luning chicken needs a systematic evaluation. In this study, we identified candidate genes regulating body size traits in Luning chicken with a genome-wide association study. These findings help to confirm the genetic mechanisms related to growth of Luning ‘yellow feather’ chickens, which should aid the development of breeding programs.

## 1. Introduction

Chickens are one of the most economically important animals in the world with a population of over 34.4 billion in 2023 according to Food and Agriculture Organization (FAO) statistics [1]. The broiler industry has evolved significantly over the past century, with a focus on rapid growth rates, efficient feed conversion, and high meat yield [2]. Chicken is the second most numerous meat product in China, and mainly comes from white-feather and yellow-feather chickens. With the development of breeding technology, marker-assisted selection (MAS) and genomic selection (GS) have been increasingly utilized in the broiler industry to improve breeding efficiency and enhance target traits. Single-nucleotide polymorphisms (SNPs) and causal genes across the genome can be utilized to accurately estimate each chicken’s genetic potential for specific traits. Therefore, the identification of candidate genetic markers and genes would allow for more accurate and efficient selection of breeding stock, leading to faster genetic improvement and ultimately enhancing the quality and yield of broiler meat for consumers.

Chicken growth traits are well known for their genetic architectural complexity [3,4,5]. Currently, there are 5339 chicken quantitative trait loci (QTL) related to the growth traits in the Animal QTL Database (https://www.animalgenome.org/cgi-bin/QTLdb/index, accessed on 24 April 2024) [6]. The accuracy and precision of locating QTL depends, in part, on the density of the linkage map created. Unfortunately, the denser the map, the more likely that false positive QTL will be detected with linkage map-based QTL methods. A more precise mapping of traits is possible with newly available genome sequences and genome-wide association studies (GWASs).

The GWAS is a powerful study design that can identify associations between genome-wide sets of genetic variations and a specific trait using genome resequencing or high-density chip technology. This methodology has generated a myriad of robust associations for a range of traits and diseases, and the number of associated variants is expected to grow steadily as GWAS sample sizes increase [7]. With the development of modern breeding technology, GWASs have also been implemented in domestic animals to identify the genetic factors associated with important economic traits [8,9,10,11]. Most of these GWASs were carried out using SNP chips due to high sequencing costs. Whole-genome sequencing (WGS) is a more efficient technology that can detect rare and undiscovered variants. It was more appropriate to explore genomic variation information by WGS with reduced costs.

**Figure 1:**
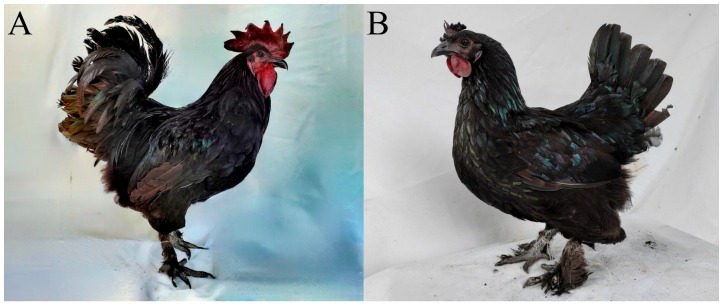
Luning chickens. Male (**A**). Female (**B**).

Luning chickens are mainly located in Mianning County, Sichuan Province, China. As a well-known native breed, Luning chickens (Figure 1) have a large body size, and exhibit well-developed chest and leg muscles, high muscle quality, high suitability, and resistance to disease [12]. The increasing market demand has driven the price of Luning chickens to more than 10 times that of ordinary broilers. All the yellow feather broilers used in production are developed from local chicken breeds. Luning chickens are an underutilized genetic resource which needs a systematic evaluation. We aim to cultivate new genetic materials that meet market and industry development needs.

In this study, GWAS analysis was performed on SNPs and INDELs by the whole-genome sequencing of 248 Luning chickens to identify potential genomic regions and explore the polygenicity of growth and development of chickens. By mapping loci associated with growth traits, it is of great significance to promote the protection, development, and utilization of chicken genetic resources and promote the development of the chicken breeding industry in China.

## 2. Materials and Methods

### 2.1. Animals and Phenotypes

The Luning chickens used in this study were raised free-range in Mianning County (location: Mianning, Sichuan, China; geographic coordinates: 101°38′~102°25′ E, 28°05′~29°02′ N, altitude: 1330~5299 m) and were obtained from the Luning chicken breeding conservation base of Sichuan Province. A total of 248 chickens were collected for the trial. All individuals were 170 days of age and allowed to eat and drink ad libitum. Blood collection and body size trait measurements were performed on the same day. Blood samples were collected by standard venipuncture into a tube with anticoagulant and stored at −20 °C for subsequent genomic DNA isolation. Eight body size traits in total were collected including body diagonal length (BDL), keel length (KL), chest width (CW), chest depth (CD), chest angle (CA), pelvic width (PW), tibial length (TL), and tibial circumference (TC), respectively. BDL, KL, CW, CD, PW, and TL were measured using a Vernier caliper, TC was measured with a tapeline, and CA was measured using a sternal protractor. To reduce the false positives caused by outliers in the association analysis, the records over three standard deviations from the mean value were removed. The animal study was reviewed and approved by the Institutional Animal Care and Use Committee of Southwest Minzu University (No. 2023069).

### 2.2. Sequencing, Quality Control, and Annotation

The genomic DNA was isolated from blood using the DNA extraction kit (CWBIO, Beijing, China). The quantity and quality of the extracted DNA were determined using a Nanodrop ND-2000 Spectrophotometer (Thermo Fisher, Waltham, MA, USA) to measure their concentration and integrity, as well as by 1% agarose gel electrophoresis to visually assess DNA integrity. DNA concentration was measured using a Qubit Flurometer (Life Technologies, Carlsbad, CA, USA)

A DNA library for each DNA sample was constructed and all qualified libraries were sequenced using the DNBSEQ-T7 sequencing platform (BGI, Shenzhen, China) with an average depth of 10×. In total, approximately 2.4 Tb of previously unpublished data were generated. To make sure the reads were reliable and contained no artificial bias in the following analyses, raw data were first processed through a series of quality control (QC) procedures. Data filtering was performed using the FASTP [13] software (version 0.19.6). Burrows-Wheeler Aligner (BWA) [14] software (version 0.7.17) was used to align the clean reads of each sample against the GCF_016699485.2_GRCg7b reference genome with the command line “BWA mem -t 4 -k 32 -M -R”. Alignment files were converted to BAM files using SAMtools (SAM) [15] software (version 1.7). In addition, the potential PCR duplications were removed using the SAMtools command “rmdup”, the pair with the highest mapping quality was only retained if multiple read pairs have identical external coordinates. Local realignment and base recalibration were performed using the Genome Analysis Toolkit (GATK) [16] software (version 3.8). The SNPs were filtered using GATK with the following criteria: QualByDepth (QD) ≥ 2.0; FisherStrand (FS) ≤ 60.0; RMSMappingQuality (MQ) < 40.0; StrandOddsRati (SOR) ≤ 3.0; MappingQualityRankSumTest (MQRankSum) ≥ −12.5; and ReadPosRankSumTest (RPRankSum) ≥ 8.0. The INDEL filtering conditions were set as follows: QD ≥ 2.0; FS ≤ 200; SOR ≤ 10.0; and RPRankSum ≥ 20.0. The variants were further filtered using PLINK [17] software (version 1.90) with the following criteria: MAF (minor allele frequency) < 0.05, Max-missing (maximum deletion rate of genotype) > 0.1, and HWE (deviations from the Hardy–Weinberg equilibrium) < 1 × 10^−6^. After filtering 18,417,354 raw SNPs and 2,866,603 INDELs, 10,186,972 SNPs and 825,511 INDELs were retained, respectively. SNP and INDEL annotation was performed according to the GCF_016699485.2_GRCg7b reference genome using ANNOVAR software (https://annovar.openbioinformatics.org/en/latest/user-guide/download/, accessed on 5 December 2023) [18].

### 2.3. Statistical Analysis

Principal component analysis (PCA) using all autosomal SNPs was performed before the association tests to estimate population stratification. A mixed linear model approach was used for GWAS, as implemented in the GMAT [19] software (https://github.com/chaoning/GMAT, accessed on 5 December 2023). The mixed linear model (MLM) [20] used in the study was acknowledged for GWAS analysis due to its effective correction of population structure and intricate intra-population relationships. The model for the GWAS was:

y = Xβ + wα + u + e

where y is the vector of phenotypic observations; β is a vector of fixed effects, including batch–sex effects and genotype at the SNP, which were fitted as covariates; X is the incidence matrix for β; w is a vector of marker genotypes; α is the effect size of the marker; u ~ N (0, Kσ^2^_a_) is the random polygenic effects; e ~ N (0, Iσ^2^_e_) is the residuals; K is the marker-derived relationship matrix; and σ^2^_a_ and σ^2^_e_ are the variances for polygenic effects and random residuals, respectively.

GWAS was carried out for the 10,186,972 retained SNPs for eight body size traits using GMAT software. The whole-genome and suggestive significance thresholds were corrected by the Bonferroni test [21] (0.05/10,186,972 and 1/10,186,972), respectively. GWAS was also carried out for the INDELs for eight traits using the same analytical process as that was used for the SNPs. The whole-genome and suggestive significance thresholds were corrected by the Bonferroni test (0.05/825,511 and 1/825,511), respectively.

The Manhattan and quantile–quantile (QQ) plots were visualized from the GWAS results by the CMplot package (https://github.com/YinLiLin/CMplot, accessed on 5 December 2023) in the R software (version 4.3.0). The SNPs and INDELs that reached the suggestive genome-wide threshold were annotated using ANNOVAR software. The genes nearest or harboring significant SNPs and INDELs were identified as candidate genes. The linkage disequilibrium (LD) correlation (r^2^) between the associated SNPs (genome-wide suggestive and significant loci on chromosome 1 and 11) was estimated and visualized by Haploview (version 4.1).

## 3. Results

### 3.1. Phenotypic Statistics, Population Structure, and SNP and INDEL Characteristics

In the study, eight body size traits of 248 Luning chickens were analyzed and the descriptive statistics including sample number, mean, standard deviation (SD), maximum (Max), minimum (Min), median, and coefficient of variation (CV) were summarized (Table 1). The CA trait had the highest CV at 29.14%, followed by PW and TL at 11.68%, and BDL had the lowest CV at 8.05%. The results indicate that there was significant variability in eight traits. Totals of 10,186,972 SNPs and 825,511 INDELs were obtained for further association analysis after quality control. All the retained SNPs (Figure S1A) and INDELs (Figure S1B) were uniformly distributed on each chromosome and mostly located in intron and intergenic regions (Figure S2). Pairwise kinship was estimated using autosomal SNP information (Figure S3), and the result indicated no population stratification effect.

### 3.2. GWAS Based on SNPs and Candidate Genes

**Figure 2:**
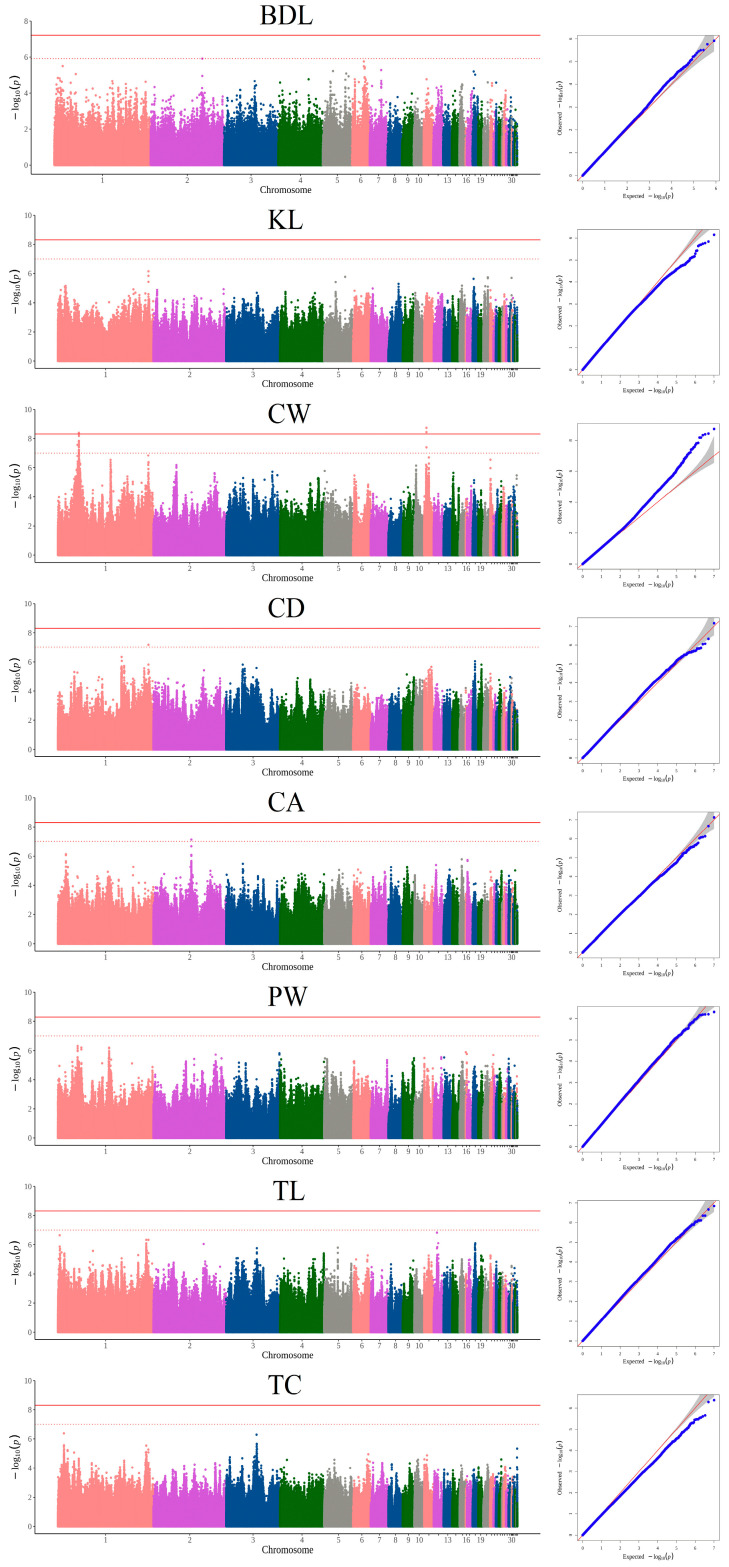
Manhattan and quantile–quantile (QQ) plots of SNP-based GWAS for 8 body size traits in Luning chickens. The Manhattan plot is on the left and the QQ plot is on the right. The horizontal solid lines and dashed lines denote the genome-wide significance (4.91 × 10^−9^) and suggestive significance thresholds (9.82 × 10^−8^), respectively.

After filtering, a total of 10,186,972 autosomal SNPs detected in 248 Luning chickens were left for GWAS. The univariate GWAS was performed on BDL, KL, CW, CD, CA, PW, TL, and TC, respectively. Manhattan and QQ plots are shown in Figure 1. The results show that 24 SNPs from 40.30 Mb to 43.22 Mb on chromosome 1 and 3 SNPs at 4.75 Mb on chromosome 11 were significantly associated with CW (Figure 2 and Table 2). The most significant SNP was at 4.75 Mb of chromosome 11 (*p* = 1.85 × 10^−9^). As shown in Table 2, 1 SNP on chromosome 1 was located in the intronic region of *PPFIA2* gene. Fourteen SNPs on chromosome 1 were located in the intronic region of *KITLG* gene. Nine SNPs on chromosome 1 were located in the intergenic region between *KITLG* and *DUSP6* genes. Three SNPs on chromosome 11 were located in the intronic region of *TOX3* gene. For CD, a significant SNP located in the intergenic region between the *MTNR1B* and *FAT3* genes was identified at 186.17 Mb of chromosome 1. The GWAS identified a significant SNP for CA, which was located in the region at 77.94 Mb on chromosome 2. The SNP was located in the exonic region of the *MARCH6* gene, which may lead to the altering of protein function. The Manhattan and QQ plots for BDL, KL, PW, TL, and TC revealed that these SNPs were not significant. The top SNPs for BDL (*p* = 1.15 × 10^−7^) and KL (*p* = 7.12 × 10^−7^) were both on chromosome 1 at position 186.17 Mb. As for PW and TC, the top SNPs were detected and were also located on chromosome 1 (PW: 40.58 Mb, *p* = 4.83 × 10^−7^; TC: 12.30 Mb, *p* = 4.22 × 10^−7^). A top SNP (*p* = 1.46 × 10^−7^) for TL was detected on chromosome 12 at position 6.75 Mb.

### 3.3. GWAS Based on INDELs and Candidate Genes

**Figure 3:**
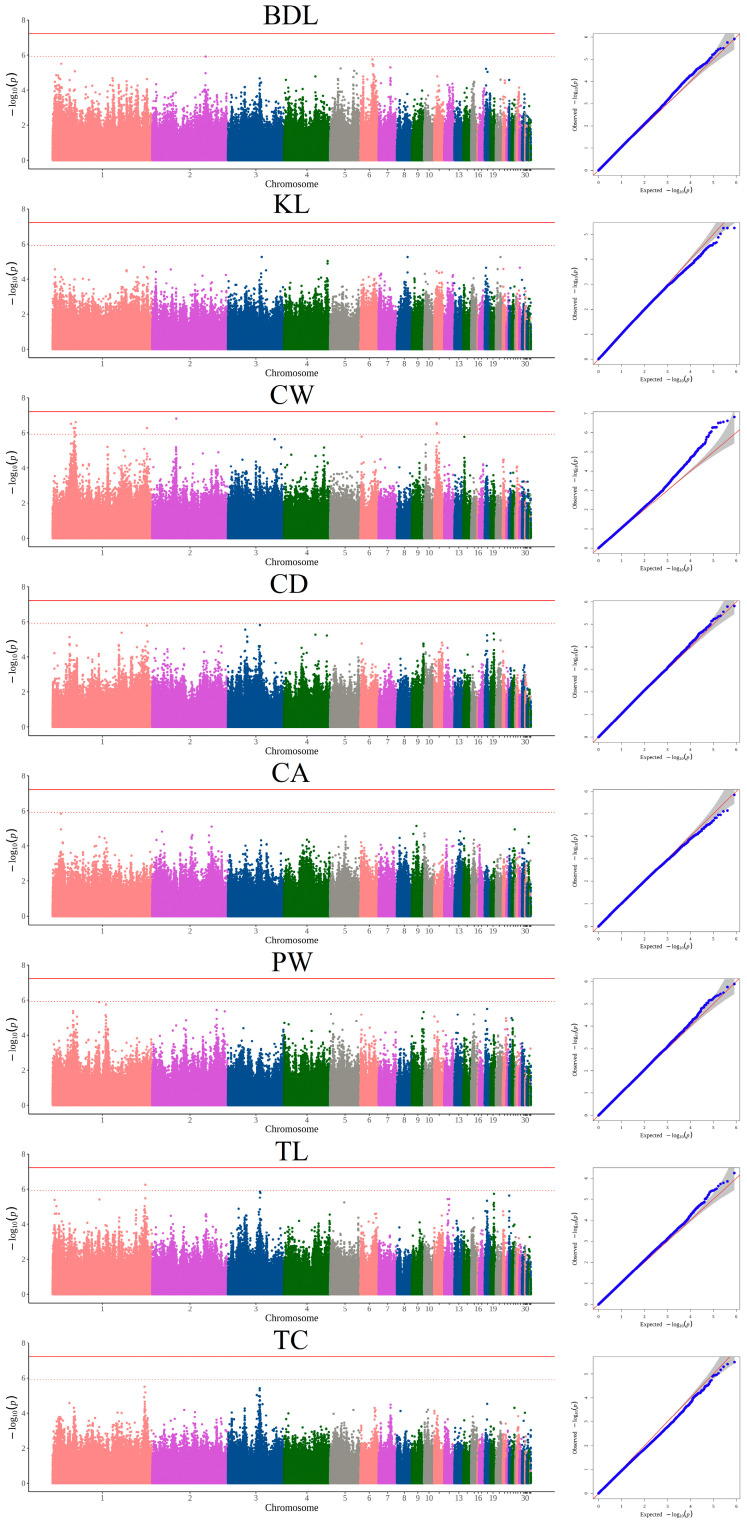
Manhattan and quantile–quantile (QQ) plots of INDEL-based GWAS for 8 body size traits in Luning chickens. The Manhattan plot is on the left and the QQ plot is on the right. The horizontal solid lines and dashed lines denote the genome-wide significance (6.06 × 10^−8^) and suggestive significance thresholds (1.21 × 10^−6^), respectively.

After filtering, a total of 825,511 autosomal INDELs detected in 248 Luning chickens were left for GWAS. The univariate GWAS was performed on 8 body size traits and identified 13 significantly associated INDELs (Figure 3 and Table 3). Twelve INDELs on chromosomes 1, 2, and 11 were significantly associated with CW and several genes were annotated including *PTPRR*, *TSPAN8*, *SLC6A15*, *TSPAN19*, *KITLG*, *DUSP6*, *POC1B*, *PLEKHG7*, *VEZT*, *MTNR1B*, *FAT3*, *BBS9*, *TOX3*, and *CYLD*. For TL, a significant INDEL located in the intergenic region between the *TRPC6* and *PGR* genes was identified on chromosome 1. Interestingly, we found that the same regions on chromosome 1 and 11 were strongly associated with CW, which implicated three and one INDELs, respectively.

### 3.4. Linkage Disequilibrium (LD) Block Analysis

A total of 25 significant SNPs on chromosome 1 were obtained for the CW trait and used to construct haplotypes. After haplotype construction with these SNPs, four smaller blocks were obtained at 43.072–43.077 Mb, 43.080–43.081 Mb, 43.083–43.085 Mb, and 43.085–43.219 Mb (Figure S4), respectively. Block 1 has four SNPs, which form two main haplotypes. Block 3 has six SNPs, which also form two main haplotypes. Ten SNPs were located in block 4, forming three major haplotypes (Figure 3). Three significant SNPs on chromosome 11 obtained for the CW trait were also used to construct haplotypes. Strong linkage disequilibrium was observed among three SNPs which formed two major haplotypes (Figure S5).

## 4. Discussion

It is well known that many genes can control the variation of more than one trait [22]. It has been shown that conducting joint analysis of multiple correlated traits could enhance the statistical power of polygenic genes and genes that only affect one of multiple related traits [23]. GWAS aims to identify the association between individual SNPs and a single trait that did not leverage information from multiple correlated traits. In our study, GMAT software was used to identify SNPs and INDELs significantly associated with body size traits in Luning chickens. The MLM has been widely applied in GWAS due to its ability to effectively control the false positive rate of SNP detection. Compared to the conventional MLM, GMAT can handle incomplete multivariate data with missing records and increase the statistical power with proper control of false positivity [24].

As an underutilized genetic resource, GWAS of Luing chickens could provide new genetic markers for white-feather and yellow-feather broilers. According to the results of GWAS, there was one SNP on chromosome 1 located in the intronic region of the *PPFIA2* gene, which belongs to the Liprin-alpha family. Liprin-alpha proteins are known to play essential roles in the regulation of synaptic function and cytoskeletal dynamics [25]. The *PPFIA2* gene was found to be significantly associated with subcutaneous fat thickness in the chicken population by GWAS [26]. This conclusion is consistent with our results which indicated that *PPFIA2* plays an important role in the regulation of body size traits. Fourteen SNPs on chromosome 1 were located in the intronic region of the *KITLG* gene. Nine SNPs on chromosome 1 were located in the intergenic region between the *KITLG* and *DUSP6* genes. Strong linkage disequilibrium was observed among 23 SNPs, which showed that the four block regions were vital to finding the true causal mutations of SNPs. KITLG is the unique ligand for the type III receptor tyrosine kinase KIT, which is also known as a stem cell factor and a mast cell growth factor [27]. The activation of KIT signaling has been found to mediate cell survival, migration, and proliferation depending on the cell type. KITLG/KIT signaling is crucial for normal hematopoiesis, pigmentation, fertility, gut movement, and some aspects of the nervous system [28]. The *KITLG* gene plays an essential role in the regulation of the litter size of goats [29,30], chicken egg production [31,32], and melanogenesis [33,34,35]. To date, there have been no reports indicating that the *KITLG* gene affects body size traits. Further research on the related mechanisms is needed in the future. DUSP6 is a member of the Mitogen-activated protein kinase (MAPK) phosphatase family, which plays a critical role as a negative regulator of the MAPK pathway [36,37]. MAPK families play an important role in complex cellular programs like proliferation, differentiation, development, transformation, and apoptosis [38]. A variant in the *DUSP6* gene correlated with body mass was identified in the QTL mapping data for body mass in the context of muscular dystrophy [39]. *DUSP6* knockout mouse models displayed dwarfism-related skeletal abnormalities [40]. *DUSP6* expression was suppressed in human and mice osteoporosis cases and *DUSP6* overexpression prevented osteoclastogenesis was confirmed in vitro experiments [41]. These data identified DUSP6 as an important regulator in skeletal development and muscle growth. Three SNPs on chromosome 11 were located in the intronic region of the *TOX3* gene and strong linkage disequilibrium was observed among three SNPs. TOX3 is identified as a calcium-dependent transactivator that exerts its effect on cAMP response element (CRE)-mediated transcription [42]. *TOX3* overexpression activates the gluconeogenic program, resulting in hyperglycemia and insulin resistance in mice and hepatocyte-specific *TOX3* knockout suppresses gluconeogenesis through FOXO1 [43]. FOXO1 could regulate skeletal muscle cell differentiation and skeletal muscle atrophy [44,45]. The *TOX3* gene can be considered to be a candidate gene for the growth and development of chickens. However, the related mechanism needs further verification.

The significant SNP associated with the CD trait on chromosome 1 was located in the intergenic region between the *MTNR1B* and *FAT3* genes. MTNR1B is one of two high-affinity forms of a receptor for melatonin, which is the primary hormone secreted by the pineal gland. It has been reported that the *MTNR1B* gene was expressed in the bone forming cells to regulate their function of depositing bone [46]. FAT3 was found to maintain the proper size of the progenitor cell pool by inhibiting the Hippo signaling pathway [47]. The Hippo pathway is influenced by metabolic, mechanical, and hormonal stimuli, which are essential mechanisms underlying the hypertrophic adaptation to resistance exercise in skeletal muscle [48]. As a crucial component of the Hippo pathway, we speculate that the *FAT3* gene could regulate the development of chicken muscle through the Hippo signaling pathway. Although these significant SNPs are located in the intronic region of the genes, they may enhance regulation by regulating the promoter. The significant SNP associated with the CA trait on chromosome 2 was located in the exonic region of the *MARCH6* genes, which changed the amino acid sequence of the MARCH6 protein. MARCH6 is an E3 ubiquitin ligase that localizes to endoplasmic reticulum (ER) membranes and plays a crucial role in homeostasis [49]. A study found that the *MARCH6* gene was differentially expressed in wild and domestic chicken breeds due to selection pressure [50]. Five SNPs of the *MARCH6* gene were identified showing strong associations with obesity by GWAS [51]. Thus, we speculate that the exonic SNP has a potential role in the development of the sternum by changing the structure of the MARCH6 protein.

A total of 13 INDELs in the GWAS analysis reached a significant level of association. Two INDELs on chromosome 1 were located in the intergenic region between *KITLG* and *DUSP6* genes, and two INDELs were located in the intronic region of the *TOX3* gene. These three candidate genes are consistent with our above results. Some genes, including *PTPRR*, *VEZT*, *BBS9*, and *CYLD*, which were found to be associated with growth traits, were considered to be candidate genes for growth traits in chickens. The PTPRR gene was identified as a candidate gene for the average daily gain trait in cattle [52]. The *VEZT* gene was found to be associated with progeny weaning weight, litter mean weight at weaning, and total litter weight at weaning in sheep by GWAS [53]. The recessive lethal 212 kb deletion in the *BBS9* gene was identified as a candidate gene affecting fertility and growth in pigs [54]. The *CYLD* gene was identified to be associated with carcass weight and intramuscular fat deposition in cattle [55]. Other genes, including *TSPAN8*, *SLC6A15*, *TSPAN19*, *POC1B*, *PLEKHG7*, *TRPC6*, and *PGR*, can be considered to be novel candidate genes for growth traits in chickens. Although these INDELs are located in the intronic and intergenic regions, they may influence gene expression by encoding non-coding RNAs, new transcripts, or regulatory elements leading to significant changes in certain traits. For example, *PGR* expression was regulated by miR-219-5p targeting a long non-coding RNA immediately downstream of the *PGR* gene [56]. Taurine upregulated gene 1 (TUG1), a long non-coding RNA, regulated the expression of *TRPC6* by modulating the TUG1–miR-145-5p–TRPC6 axis [57].

## 5. Conclusions

In this study, we performed a GWAS analysis to identify crucial SNPs and candidate genes for body size traits based on WGS data for 248 Luning chickens. A total of 30 SNPs and 13 INDELs with significant effects were detected and some genes, including *PPFIA2*, *KITLG*, *DUSP6*, *TOX3*, *MTNR1B*, *FAT3*, *PTPRR*, *VEZT*, *BBS9*, and *CYLD*, were identified as important candidate genes for growth traits in chickens. In particular, *KITLG* and *DUSP6* with 25 significant SNPs and INDELs could be considered candidate genes for the chest width trait. It is of great significance to promote the protection, development, and utilization of Luning chickens.

## Figures and Tables

**Table 1:** Descriptive statistics of body size traits in Luning chickens.

Trait	Number	Mean	SE	Max	Min	Median	CV (%)
BDL	248	21.94	0.12	25.40	17.30	22.00	8.05
KL	247	12.32	0.09	15.50	8.50	12.30	10.37
CW	242	7.47	0.05	9.35	5.55	7.52	9.21
CD	248	10.88	0.06	13.20	8.84	10.80	8.33
CA	240	45.00	1.15	85.00	11.00	45.00	29.14
PW	248	6.77	0.06	9.09	4.50	6.73	11.68
TL	248	9.57	0.05	12.30	7.36	9.45	11.68
TC	248	4.57	0.03	6.00	3.20	4.60	11.26

Body diagonal length (BDL, cm), keel length (KL, cm), chest width (CW, cm), chest depth (CD, cm), chest angle (CA, deg), pelvic width (PW, cm), tibial length (TL, cm), tibial circumference (TC, cm), maximum (Max), minimum (Min), standard error (SE), coefficient of variation (CV).

**Table 2:** Significant SNPs and candidate gene information.

Trait	Chromosome	Position (bp)	Alleles	*p*-Value	Annotation	Gene Name
CW	1	40,298,901	G/A	2.70 × 10^−8^	Intronic	*PPFIA2*
	1	43,072,056	C/T	6.24 × 10^−8^	Intronic	*KITLG*
	1	43,073,484	A/G	6.06 × 10^−8^	Intronic	*KITLG*
	1	43,075,104	C/T	3.09 × 10^−8^	Intronic	*KITLG*
	1	43,077,685	C/T	4.05 × 10^−9^	Intronic	*KITLG*
	1	43,079,649	T/C	8.08 × 10^−8^	Intronic	*KITLG*
	1	43,080,517	A/C	1.51 × 10^−8^	Intronic	*KITLG*
	1	43,081,408	G/C	4.61 × 10^−9^	Intronic	*KITLG*
	1	43,083,179	C/A	2.61 × 10^−8^	Intronic	*KITLG*
	1	43,084,274	G/T	7.15 × 10^−8^	Intronic	*KITLG*
	1	43,084,312	A/G	7.98 × 10^−8^	Intronic	*KITLG*
	1	43,084,631	T/C	3.48 × 10^−8^	Intronic	*KITLG*
	1	43,084,657	T/C	1.61 × 10^−8^	Intronic	*KITLG*
	1	43,084,806	G/A	2.02 × 10^−8^	Intronic	*KITLG*
	1	43,085,497	T/C	3.87 × 10^−8^	Intronic	*KITLG*
	1	43,202,979	A/G	6.65 × 10^−8^	Intergenic	*KITLG*, *DUSP6*
	1	43,207,402	C/G	8.53 × 10^−8^	Intergenic	*KITLG*, *DUSP6*
	1	43,207,404	C/T	8.53 × 10^−8^	Intergenic	*KITLG*, *DUSP6*
	1	43,207,758	T/C	6.56 × 10^−9^	Intergenic	*KITLG*, *DUSP6*
	1	43,207,760	A/G	6.56 × 10^−9^	Intergenic	*KITLG*, *DUSP6*
	1	43,208,364	G/T	6.44 × 10^−8^	Intergenic	*KITLG*, *DUSP6*
	1	43,208,452	A/T	1.45 × 10^−8^	Intergenic	*KITLG*, *DUSP6*
	1	43,208,537	G/A	3.74 × 10^−8^	Intergenic	*KITLG*, *DUSP6*
	1	43,219,314	C/T	5.83 × 10^−8^	Intergenic	*KITLG*, *DUSP6*
	11	4,751,386	A/T	1.85 × 10^−9^	Intronic	*TOX3*
	11	4,751,411	C/T	3.65 × 10^−9^	Intronic	*TOX3*
	11	4,751,668	A/C	3.96 × 10^−8^	Intronic	*TOX3*
CD	1	186,174,672	G/A	6.82 × 10^−8^	Intergenic	*MTNR1B*, *FAT3*
CA	2	77,942,520	C/T	7.43 × 10^−8^	Exonic	*MARCH6*

Chest width (CW, cm), chest depth (CD, cm), chest Angle (CA, deg).

**Table 3:** Significant INDELs and candidate gene information.

Trait	Chromosome	Position (bp)	Alleles	*p*-Value	Annotation	Gene Name
CW	1	36,193,064	G/GCA	2.96 × 10^−7^	Intergenic	*PTPRR*, *TSPAN8*
	1	41,770,569	GAGAA/G	5.15 × 10^−7^	Intergenic	*SLC6A15*, *TSPAN19*
	1	43,198,933	T/TA	8.75 × 10^−7^	Intergenic	*KITLG*, *DUSP6*
	1	43,206,303	C/CACT	9.98 × 10^−7^	Intergenic	*KITLG*, *DUSP6*
	1	43,376,793	C/CGTGGATCTGA	5.37 × 10^−7^	Intergenic	*DUSP6*, *POC1B*
	1	44,656,419	AT/A	5.11 × 10^−7^	Intronic	*PLEKHG7*
	1	45,559,065	AT/A	2.39 × 10^−7^	Intronic	*VEZT*
	1	186,174,628	A/AGGCAGCT	5.22 × 10^−7^	Intergenic	*MTNR1B*, *FAT3*
	2	47,680,889	AAC/A	1.52 × 10^−7^	Intronic	*BBS9*
	11	4,751,536	G/GATA	2.73 × 10^−7^	Intronic	*TOX3*
	11	4,799,561	CAG/C	3.12 × 10^−7^	Intronic	*TOX3*
	11	5,847,690	TCGA/T	1.04 × 10^−6^	Intronic	*CYLD*
TL	1	183,023,926	C/CT	5.63 × 10^−7^	Intergenic	*TRPC6*, *PGR*

Chest width (CW, cm), tibial length (TL, cm).

## Data Availability

The original contributions presented in the study are included in the article/Supplementary Materials, further inquiries can be directed to the corresponding author.

## Supplementary Materials

The following supporting information can be downloaded at: www.mdpi.com/article/10.3390/ani15070972/s1, Figure S1. Distribution of SNPs and INDELs on each chromosome; Figure S2. Distribution of SNPs and INDELs on chromosome position; Figure S3. The population structure of Luning chickens evaluated by the first three principal components; Figure S4. LD blocks with 25 significant SNPs on chromosome 1 that affect the CW traits; Figure S5. LD blocks with 3 significant SNPs on chromosome 1 that affect the CW traits.
