# Supplementary material for: Genome-Wide Association Study of Body Size Traits in Luning Chickens Using Whole-Genome Sequencing
Source: Animals (Basel). 2025 Mar 27;15(7):972. doi: 10.3390/ani15070972 (PMC11987916; doi:10.3390/ani15070972)
Supplement: Supplementary file 1 [file animals-15-00972-s001.zip › animals-3401653-supplementary.pdf]

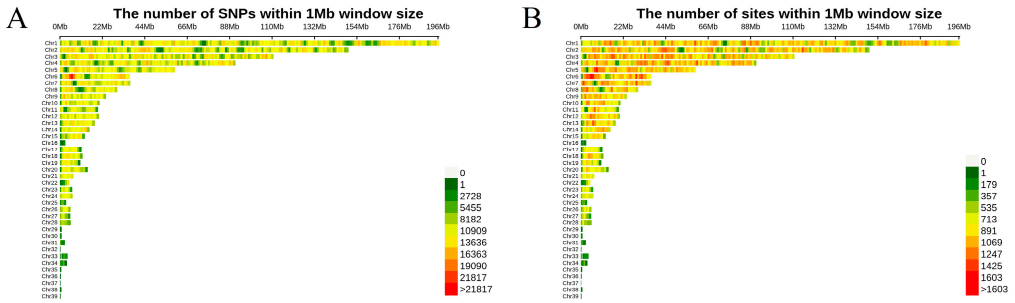

Figure S1. Distribution of SNPs and INDELs on each chromosome. (A) Distribution of SNPs on each chromosome. (B) Distribution of INDELs on each chromosome.

A

B

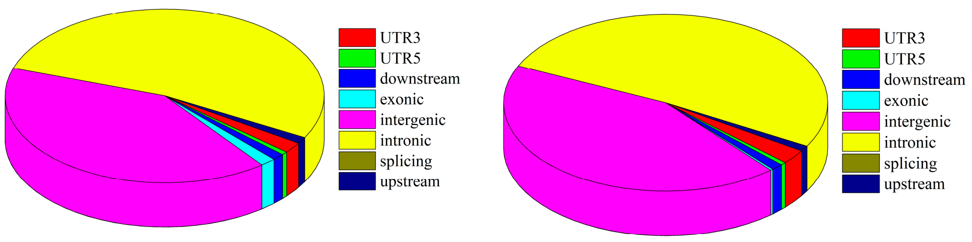

Figure S2. Distribution of SNPs and INDELs on chromosome position. (A) Distribution of SNPs on chromosome position. (B) Distribution of INDELs on chromosome position.

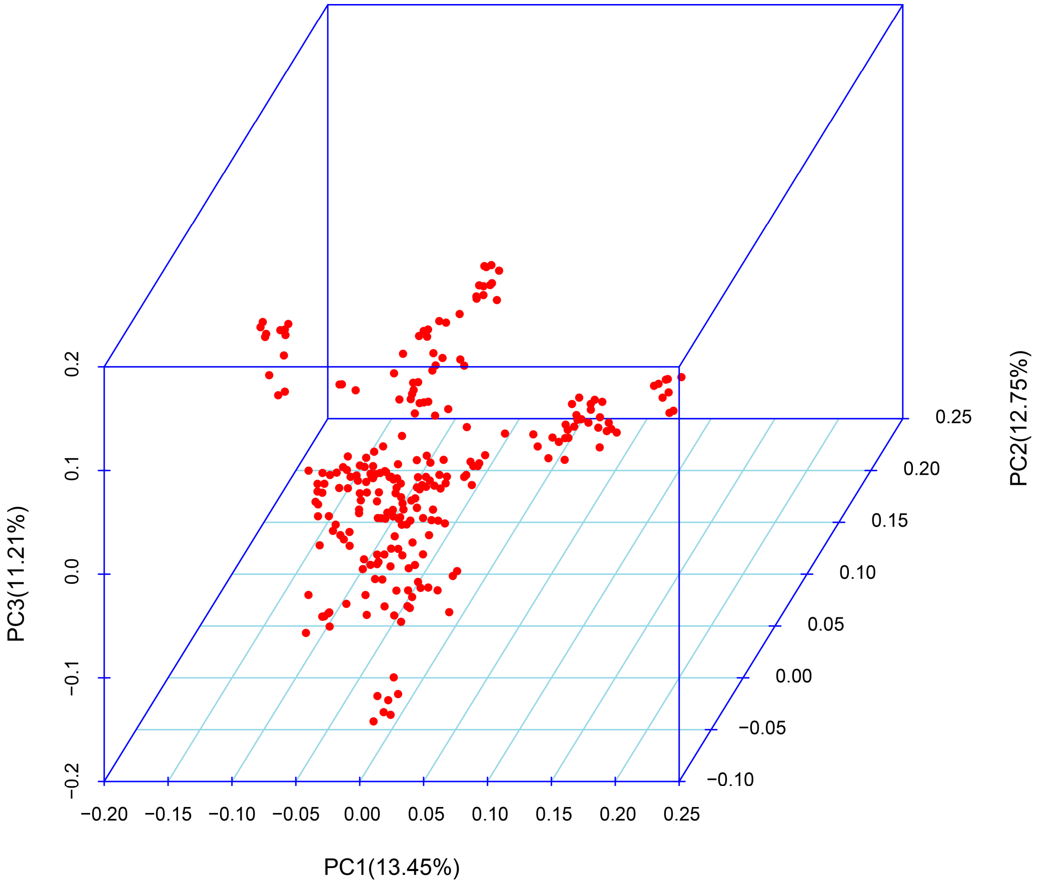

Figure S3. Population structure of Luning chicken evaluated by the first three principal components.

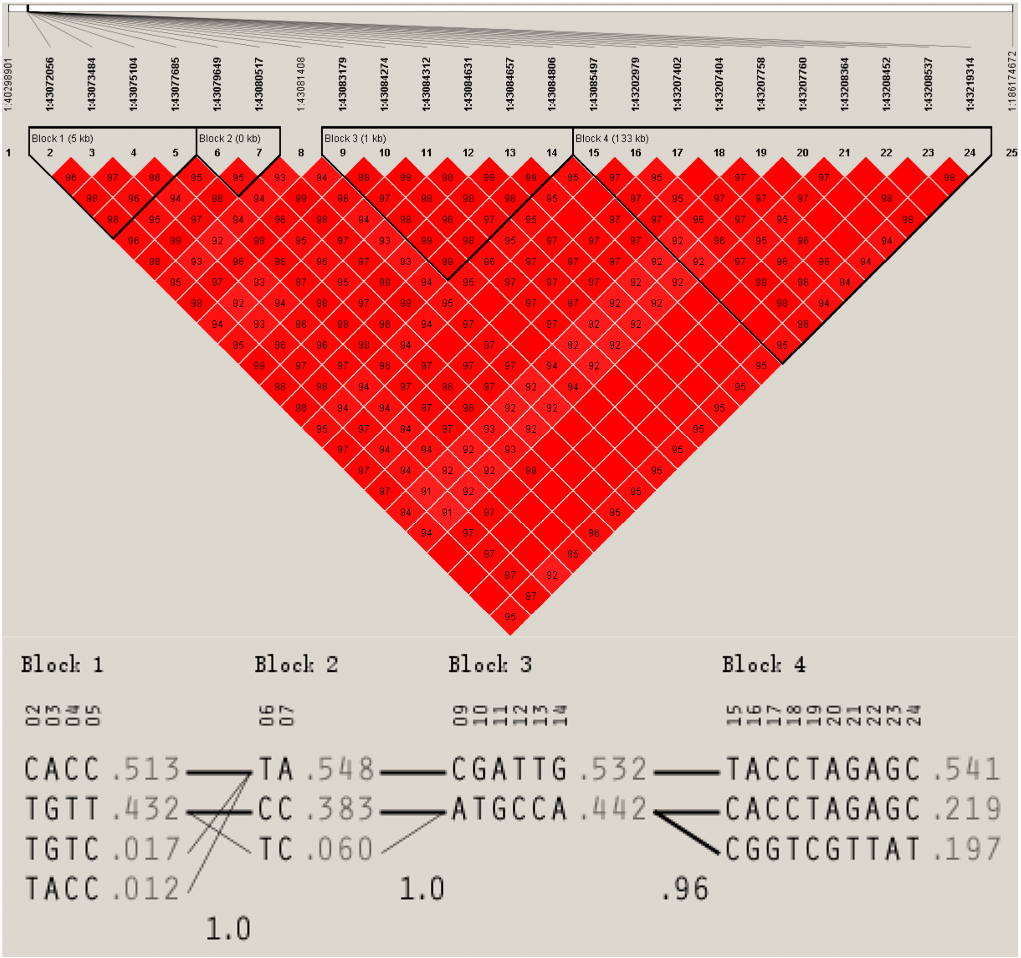

Figure S4. LD blocks with 25 significant SNPs on chromosome 1 that affect the CW traits. Four blocks containing multiple SNPs were obtained.

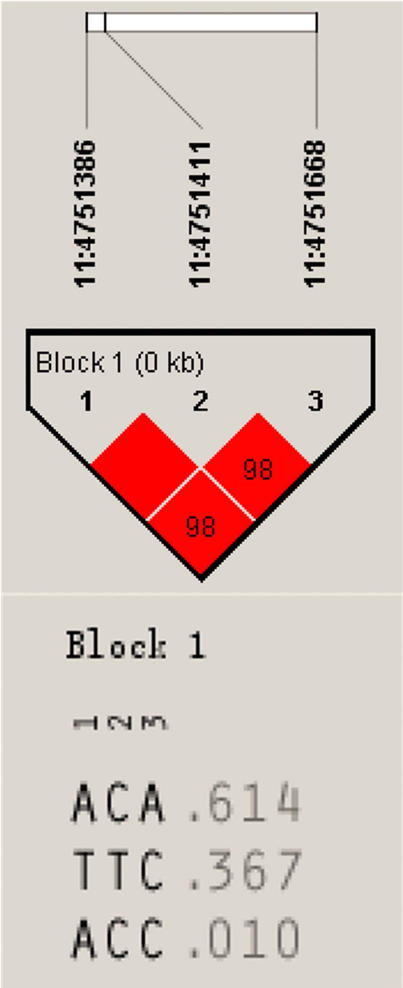

Figure S5. LD blocks with 3 significant SNPs on chromosome 1 that affect the CW traits. One block containing three SNPs was obtained.
